# Family-Based Multi-SNP X Chromosome Analysis Using Parent Information

**DOI:** 10.3389/fgene.2016.00020

**Published:** 2016-02-22

**Authors:** Alison S. Wise, Min Shi, Clarice R. Weinberg

**Affiliations:** Biostatistics and Computational Biology Branch, National Institute of Environmental Health SciencesDurham, NC, USA

**Keywords:** X chromosome, case-parent triad, maternally-mediated effects, haplotype analysis, oral cleft

## Abstract

We propose a method for association analysis of haplotypes on the X chromosome that offers both improved power and robustness to population stratification in studies of affected offspring and their parents if all three have been genotyped. The method makes use of assumed parental haplotype exchangeability (PHE), a weaker assumption than Hardy-Weinberg equilibrium (HWE). PHE requires that in the source population, of the three X chromosome haplotypes carried by the two parents, each is equally likely to be carried by the father. We propose a pseudo-sibling approach that exploits that exchangeability assumption. Our method extends the single-SNP PIX-LRT method to multiple SNPs in a high linkage block. We describe methods for testing the PHE assumption and also for determining how apparent violations can be distinguished from true fetal effects or maternally-mediated effects. We show results of simulations that demonstrate nominal type I error rate and good power. The methods are then applied to dbGaP data on the birth defect oral cleft, using both Asian and Caucasian families with cleft.

## Introduction

Haplotype analysis can be more powerful than single-SNP analysis because haplotypes can capture multi-marker information simultaneously (Akey et al., 2001; Morris and Kaplan, 2002). Sets of linked SNPs within a gene's coding region can also have a joint effect on the structure of the protein product, so such analyses can potentially contribute to our understanding of mechanisms of effect. When studying autosomal haplotypes, only the unphased genotypes (the aggregate of the two haplotypes) can typically be measured. For certain genotypes it is consequently impossible to identify the two haplotypes (for example if a person is heterozygous at more than one of the loci). Therefore, for both family-based studies and population-based association studies, methods have been developed to account for phase ambiguity (Clayton, 1999; Chung et al., 2006; Lin and Zeng, 2006; Allen and Satten, 2007) in haplotype analyses of the autosome. For nuclear family-based methods, the TRIad Multi-Marker method (TRIMM), introduced by (Shi et al., 2007) allows for haplotype analysis without phase assignment and TRIMMest (Shi et al., 2009) extends the method to enable estimation of the relative risk for a candidate haplotype.

By contrast, the X chromosome is unique and wonderfully cooperative in that, as we will show, when complete case-parents genotype data is present there is no phase ambiguity. Methods currently available to analyze haplotypes on the X chromosome in nuclear families are the X-LRT (Zhang et al., 2008), the X-APL (Chung et al., 2007), UNPHASED (Dudbridge, 2008), and HAPLIN (Gjessing and Lie, 2006; Jugessur et al., 2012). UNPHASED and HAPLIN were originally developed to analyze variants on the autosome. HAPLIN, X-LRT, and UNPHASED are all likelihood-based methods that provide estimates of the haplotype relative risks, relative to a reference haplotype. X-APL cannot provide haplotype relative risk estimates but X-APL and UNPHASED were designed to fully use information for nuclear families with one or more affected siblings. HAPLIN was designed for case-parent triads but can also be used for case-control data and case-parent/control-parent triads. X-LRT analyzes case-parent triads, and can use sibling data to help inform the analysis in the presence of missing genotypes. However, the X-LRT method is limited to two-marker haplotypes, and we will not consider it further here. FBAT is a nonparametric method based on conditioning on parental genotypes. It should be noted that, if one can assume Hardy-Weinberg equilibrium (HWE), all five of these methods can account for missing genotype data. Currently, the method we will present only handles complete triads, but it offers robustness because HWE is not required.

PIX-LRT (the parent-informed X chromosome likelihood ratio test; Wise et al., 2015) is a method to measure individual SNP effects of fetally-inherited X chromosome variants. PIX-LRT offers improved power by using both the information from transmission of a variant X allele from parents to affected offspring, and information from the distribution of the parental genotypes across the mother/father pairs.

An assumption of “parental allelic exchangeability” (Shi et al., 2008) enables added parental information to be captured in a way that resists bias due to genetic population stratification. Parental exchangeability is here adapted to apply to sets of SNPs on the X chromosome that are in a region of low recombination, as follows: we assume that in the source population at large the three haplotypes (SNP allele sets) carried by each pair of parents are randomly distributed between them, two to the mother and one to the father. Note that parental haplotype exchangeability (PHE) is a far weaker assumption than HWE, implying broader applicability of the method. Under exchangeability, HWE may not hold. For example, the population may include incompletely admixed subpopulations, with varying prevalences of particular risk haplotypes and varying baseline susceptibility to the disease, as in population stratification. But within each subpopulation there can nonetheless be random allocation of the haplotypes to the two parents for each couple. Under this exchangeability assumption and in the absence of maternally-mediated effects we generalize PIX-LRT to allow association studies of haplotype effects for the X. The PHE assumption can be tested, as will be demonstrated.

In the following sections, we describe “PIX-HAP,” an extension of PIX-LRT for testing effects of X haplotypes based on case-parent triads. We compare the performance of PIX-HAP to that of HAPLIN, UNPHASED, FBAT, and X-APL using simulations to assess Type I error rates and power. As an illustrative example, we apply PIX-HAP to data from a family-based oral cleft dataset to analyze haplotypes on the X chromosome. Testing the PHE assumption serves to expose some genotyping issues and identifies a haplotype that might act indirectly through maternal effects. We conclude with a discussion of the advantages and limitations of using PIX-HAP to study haplotypes.

## Subjects and methods

### Case-parent design and assumptions

We consider a sample of genotyped case-parent triads, where all the genotyped offspring have been diagnosed with the condition of interest. For fathers and sons, the haplotype is directly measured, as fathers and sons both have just one X chromosome. We represent a haplotype as a vector of 0 s and 1 s, with 1 indicating the presence of the minor allele at the corresponding locus. For mothers and daughters, the measurable genotype is the phase-ambiguous summed combination of the two haplotypes from their X chromosomes. We can nonetheless identify the individual haplotypes in females if we assume no recombination between the variants considered. To see why, consider that each female offspring has inherited her father's complete X. If we subtract the father's contribution from the daughter's genotype, we can infer the daughter's maternally-inherited haplotype at any linked set of loci. Hence we can also identify the two haplotypes carried by the mother, by subtracting that inferred transmitted haplotype from the summed maternal genotype. For triads with male offspring (who only have one haplotype, which is always maternally-derived) we also know exactly the two haplotypes carried by the mother, again by subtraction. Thus, unlike for the autosome, for the X we can identify the three haplotypes carried by the parents and specify which were transmitted to the affected offspring.

We assume there is Mendelian transmission of the haplotype in the source population. We also assume PHE in the source population: within a random mating pair, at the location of the haplotype, the three haplotypes carried by the two parents are randomly allocated between the father's single chromosome and the mother's two chromosomes. We also assume that the variants are not determinants of fetal survival or parental ability to reproduce. As before, we exclude consideration of the pseudo-autosomal regions and the X-transposed region (PARs, XTR), as these regions on the X can meiotically cross over with a homologous region on the Y.

### PIX-LRT extension to haplotype analysis

**Figure 1:**
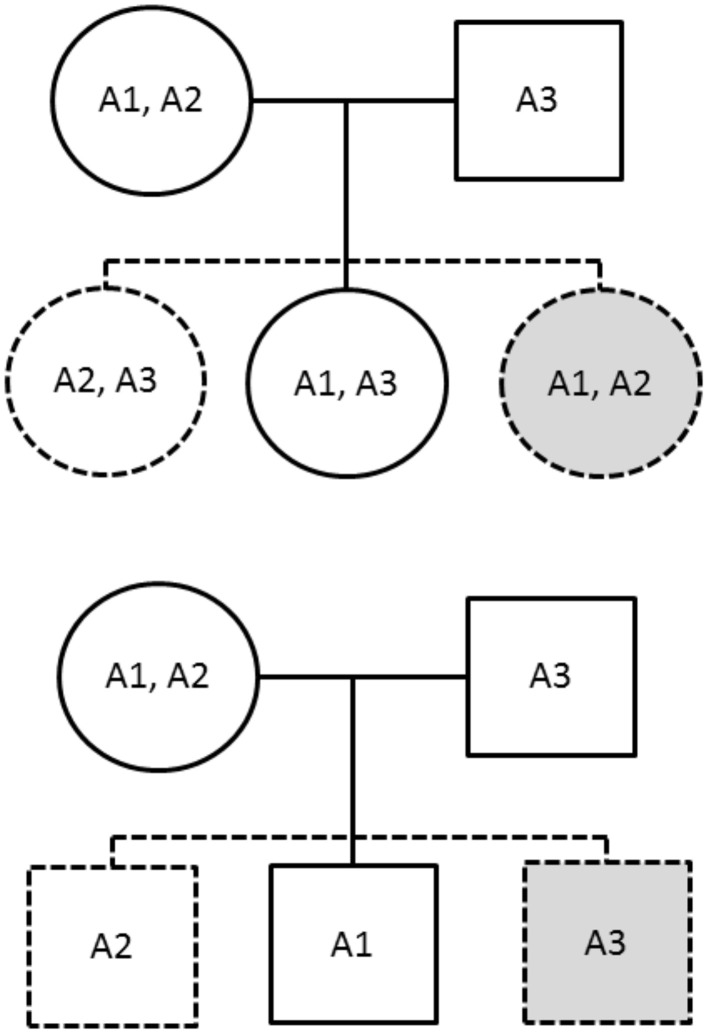
**The solid figures correspond to observed genotypes, while the dashed figures correspond to pseudo-siblings**. The upper family has an affected daughter while the lower has an affected son. The gray shading indicates additional siblings provided by imposing the parental haplotype exchangeability assumption.

The PIX-HAP method draws from the notion of pseudo-siblings as applied to haplotypes (Self et al., 1991). In our haplotype analysis, we compare for each affected child the transmitted haplotype(s) to those of two equally likely (under the null and under PHE) pseudo-siblings, where we condition on the set of three haplotypes carried by the parents. For male offspring, there are two pseudo-brothers, one carrying the nontransmitted maternal haplotype and the other carrying the father's single haplotype. For female offspring, there are two pseudo-sisters, who each carry a combinatoric pair of the observed parental X haplotypes. The family is noninformative if and only if all three haplotypes are the same. To see why PHE will enhance power, note the additional hypothetical equally-likely pseudo-siblings shown in gray in Figure 1.

While this paper will focus for simplicity on complete triad data, one can include information from families with a missing parent if the affected offspring is male. For example, if the affected child is a boy and the father is missing, the nontransmitted maternal X haplotype can serve as a single pseudo-brother. If it is instead the mother who is missing, one can use the paternal haplotype as a pseudo-brother. If a parent of a daughter case is missing, there are no obvious corresponding remedies without additional assumptions. For example, the paternal genotype must be known, to phase the mother.

We use a conditional logistic regression model in R for this analysis. Conditioning is on the family and the 3 haplotypes carried by the parents. The pseudo-offspring who serve as controls are the possible other children of the same sex produced by those 3 haplotypes. The R function (“PIX-HAP”) is available for downloading from the NIEHS website.

Families with male versus female offspring can be analyzed separately, so that no quantitative relationship between the relative risks in the male offspring and female offspring needs to be imposed. To simplify our analysis, we model the haplotype risk in males to be the same as the risk in females who carry two copies of the haplotype. To accommodate rare haplotypes, we impose a threshold based on the parental frequencies. Haplotypes with frequencies beneath that designated threshold are grouped together, with the associated relative risk treated as a nuisance parameter with little meaning. For example if two or more haplotypes have frequencies beneath the threshold, those haplotypes are combined into a single aggregated “haplotype.” The likelihood is then calculated twice: once under an alternative to the null with a separate coefficient for each haplotype allowed in the model including the aggregated haplotype, and again as the null, with only a coefficient for the aggregate rare “haplotype” in the model, in effect pooling together all the others that are not rare as equivalent. The likelihood ratio test statistic is calculated by calculating the change in the deviance for the former model compared to the latter model. We have set the haplotype prevalence threshold at 0.01 for our simulation studies.

To consider as our potential haplotypes sets of 4 SNPs taken in order by location, we slide a 4-locus window across the X chromosome, capturing overlapping sets of neighboring SNPs. Although there are potentially 16 (=2^4^) haplotypes for each such window, requiring a 15 degree-of-freedom test, typically many will be rare and the degrees of freedom for the chi-squared likelihood ratio test will be much lower than 16.

### Type I error and power calculations

We use simulations to compare X-APL, UNPHASED, HAPLIN, FBAT, and PIX-HAP under null scenarios with and without HWE. We are interested in testing the global null that no nonrare haplotype is associated with the disease, compared to an alternative where at least one nonrare haplotype is positively or negatively associated with the disease. We carry out a chi-squared test, where the degrees of freedom equal the number of nonrare haplotypes minus one. The default for HAPLIN is to remove families that carry any haplotypes that have a frequency less than 1%. X-APL and UNPHASED also have an option to remove families with rare haplotypes from their global test calculation. For comparability we set these thresholds to 1% as well. This option has the potential to increase the power to detect an effect (provided the susceptibility haplotype has a prevalence above 1%), as it can decrease the number of tests, and hence the degrees of freedom. HAPLIN also allows the user to specify the relationship between the effect of a male carrying one copy of the variant and a female carrying one or two copies. In analyses of our simulations with HAPLIN we set its “comb.sex” option to “double,” which sets the effect of males having one copy of the variant equal to that for females having two, an approximation that can be seen as adjusting for X inactivation. This setting optimizes the performance of both HAPLIN and PIX-HAP, as it coincides with the model used in producing the simulations, whereas in practice one would not know the actual relationship.

For the null simulation, we simulated scenarios that involve four markers and 16 haplotypes. Scenario 1 in Table 1 shows the haplotype frequencies used for the null scenario simulations under HWE. Each dataset contained 1000 families and we simulated 5000 datasets. For simplicity we assumed that males and females have the same risk of disease. To simulate a scenario in which HWE is violated, we mixed two genetically different subpopulations with the haplotype frequencies of subpopulations 1 and 2 of Table 1. The risk of disease in the second population was four times that in the first population. Again we ran 5000 simulations, each based on 1000 families.

**Table 1:** **Haplotype frequencies for the different scenarios used in the simulations**.

**Haplotype**	**Frequencies**	
	**Subpopulation 1**	**Subpopulation 2**
0000	0.2401	0.4096
0001	0.1029	0.1024
0010	0.1029	0.1024
0011	0.0441	0.0256
0100	0.1029	0.1024
0101	0.0441	0.0256
0110	0.0441	0.0256
0111	0.0189	0.0064
1000	0.1029	0.1024
1001	0.0441	0.0256
1010	0.0441	0.0256
1011	0.0189	0.0064
**1100**	**0.0441**	**0.0256**
**1101**	**0.0189**	**0.0064**
1110	0.0189	0.0064
1111	0.0081	0.0016

*For the haplotype notation, “1” refers to the SNP in the haplotype carrying the minor allele. For example, “1100,” is the haplotype where the first two SNPs carry the minor allele, and the second two SNPs do not. When a risk haplotype (or two) is involved, haplotype “1100” (and “1101”) shown in bold, is the risk haplotype(s)*.

To evaluate power, again we considered 4-SNP scenarios. We simulated 1000 data sets to estimate the power at alpha level 0.05, under a range of alternative scenarios. We assumed HWE (with frequencies as for subpopulation 1 of Table 1) and again simulated 1000 families in each data set. We considered three risk scenarios (referred to as A–C). In all risk scenarios, we designated haplotype “1100” to be the risk haplotype. For this haplotype, the relative risk associated with disease for a boy carrying the risk haplotype compared to the other haplotypes (*R*_*B*_) was set at 1.5. For girls, we assumed a log-additive model: the relative risk for disease for a girl carrying one copy of the risk haplotype compared to the nonrisk haplotypes (*R*_*G*1_) was the square root of 1.5 and for a girl carrying two copies (*R*_*G*2_) was 1.5. In scenario A, only haplotype “1100” conferred risk. In B, in addition to haplotype “1100” described above haplotype “1101” also conferred risk (*R*_*B*_ = 1.2, $R_{G1}=\sqrt{1.2}$, and *R*_*G*2_ = 1.2). In scenario C, while haplotype “1100” conferred risk, haplotype “1101” conferred a protective effect (*R*_*B*_ = 1∕1.2, $R_{G1}=\left(1∕\sqrt{1.2}\right)$, and *R*_*G*2_ = 1/1.2).

We initially set our haplotype frequencies to be the same as the null situation under HWE above (scenario 1 in Table 1). We evaluated statistical power under a range of risk haplotype frequencies: 0.05, 0.1, 0.2, 0.3, 0.4, 0.5, 0.6, 0.7, 0.8, and 0.9. In scenarios B and C, in which there were two haplotypes with an effect on risk, we let the sum of their frequencies be the above, and kept the frequency ratio the same (“1100”: “1101” is 0.0441:0.0189). After modifying the risk haplotype(s) frequency, we rescaled the remaining haplotypes so the sum of all frequencies equaled 1.

### Oral cleft data

We applied PIX-HAP to the X chromosome data from the International Consortium to Identify Genes and Interactions Controlling Oral Clefts (http://www.ncbi.nlm.nih.gov/projects/gap/cgi-bin/study.cgi?study_id=phs000094.v1.p1#attribution-section). A complete haplotype analysis has not previously been performed on this dataset; however, (Patel et al., 2013) previously analyzed selected X SNPs using those data. They used UNPHASED (Dudbridge, 2008) to analyze combinations of the 25 SNPs in the Duchenne muscular dystrophy (DMD) gene, because individual SNPs in that gene had shown strong associations for the phenotype cleft lip with or without cleft palate (CL/P).

Here we restrict our analysis to complete triads of either Asian (including Pacific Islanders) or Caucasian ethnicities. We analyzed Asian and Caucasians family triads both separately and combined. Additionally, we tested haplotypes separately for cleft palate only (CPO) and cleft lip with or without palate (CL/P), based on evidence that those two phenotype categories have distinct genetic etiologies (Murray, 2002). The gender and cleft subtype breakdown is shown in Table 2. Notice that the two phenotypes differ in the sex ratio of affected offspring.

**Table 2:** **Complete case-parent families by cleft type, gender and ancestry**.

	**European**		**Asian**		**Total**	
	**Male**	**Female**	**Male**	**Female**	**Male**	**Female**
Cleft Type						
CL/P	424	240	575	312	999	552
CPO	105	107	93	140	198	247
Total by gender	529	347	668	452	1197	799
Total	876		1120		1996	

*CL/P is cleft lip with or without palate, CPO is cleft palate only*.

We used a sliding window approach to analyze haplotypes, by analyzing in turn successive overlapping sets of 4 neighboring (in the panel available) SNPs on the X chromosome. We first filtered by considering only those SNPs with a minor allele frequency in parents greater than 0.05, and also restricting to those with a unique mapping from the Illumina Human610-Quad v1.0 Build 36 to Build 37. We also excluded SNPs for which we had genotyping concerns (rs17269319, rs3747355, rs5906541, rs12558269). As pointed out in an earlier paper (Wise et al., 2015), no father carried a variant of any of these, despite some missing fathers having evidently transmitted a copy to their daughter. There were 10,571 SNPs that passed this screening among Asians, 12,417 SNPs amongst Caucasians, and 12,365 SNPs amongst the combined populations. For 4-SNP moving window haplotype analyses, the number of haplotype tests is then the total number of SNPs minus 3. The appropriate alpha levels for a Bonferroni-corrected family-wise error rate of 0.05 for Asians, Caucasians and the combined sample are consequently 4.73 × 10^−6^, 4.03 × 10^−6^, and 4.04 × 10^−6^, respectively.

We considered a *p*-value to be significant if it was lower than the Bonferroni-corrected *p*-value. To display results, we constructed plots of −log_10_(*p*-value) against the marker position of the first SNP in the haplotype along the X chromosome (as determined by human genome Build 37).

We use the same sliding windows to test the assumption of PHE. We did this test by fitting a conditional logistic model where the father's X haplotype is treated as the “case” and the two carried by the mother are treated as controls. This test of PHE is valid under the global null that there are no effects of the haplotypes under consideration, and under the assumption that there are no maternally-mediated effects. Rejection of the null can happen for reasons other than violation of PHE in the population, as will be discussed in our data example below.

## Results

### Simulation results

Under a null scenario of no association between the haplotype and disease, for scenarios simulated under HWE, all methods maintained the nominal type I error rates except for X-APL. When there was population stratification and HWE was violated, PIX-HAP, FBAT, and HAPLIN again had type I errors close to the nominal levels while UNPHASED and X-APL both appeared to have inflated type I error (Table 3).

**Table 3:** **Simulated Type I error rates for X-haplotype methods**.

	**PIX-HAP**	**HAPLIN**	**X-APL**	**UNPHASED**	**FBAT**
HWE	0.050	0.050	0.066	0.057	0.047
NO HWE	0.053	0.055	0.064	0.074	0.046

*5000 datasets simulated when Hardy-Weinberg equilibrium is correctly assumed (HWE) and when it is violated (no HWE) with 1000 triads. The 95% prediction interval for a rate of 0.05 with a sample of size 5000 is (0.044, 0.056)*.

**Figure 2:**
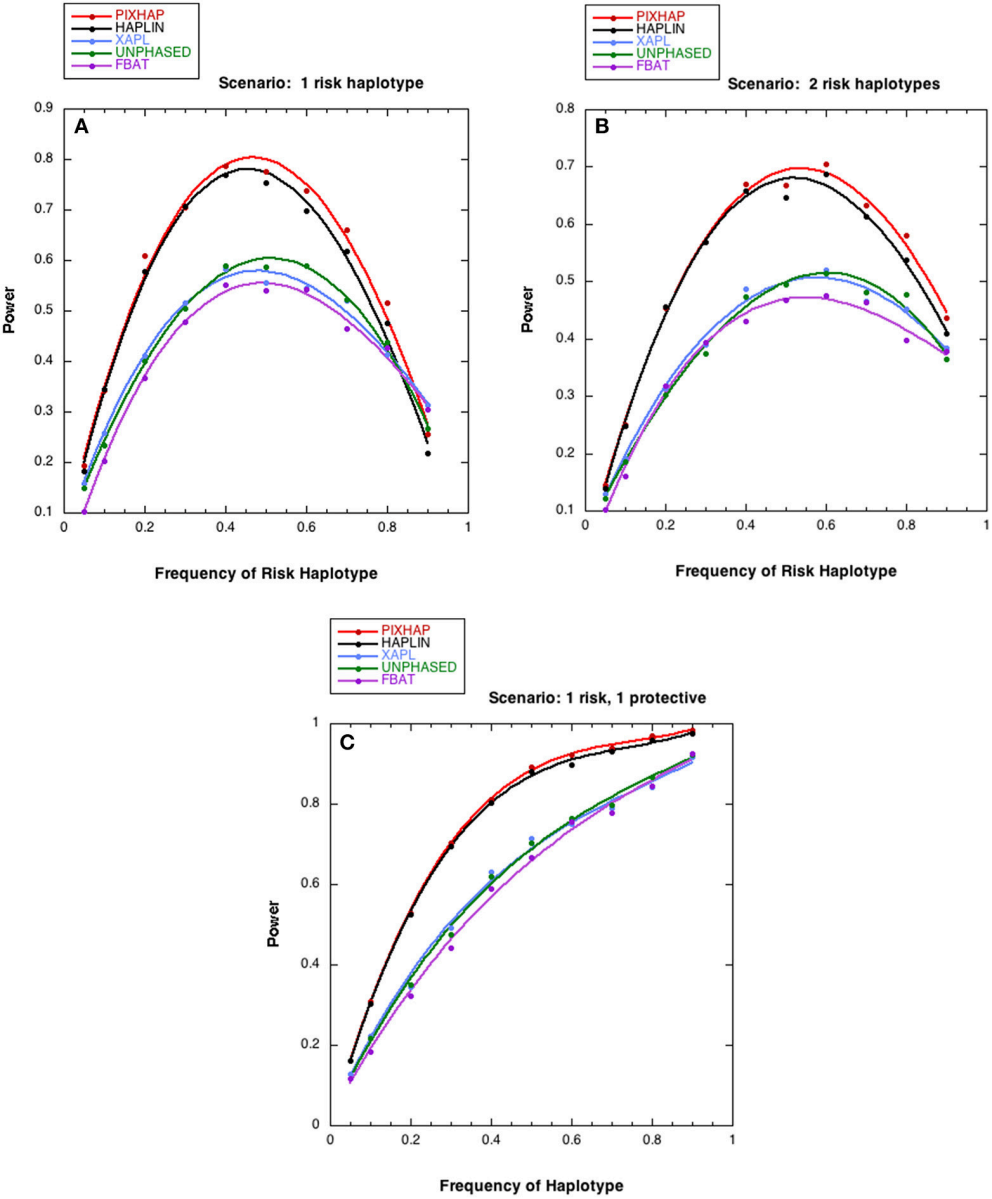
**Power estimates as a function of risk haplotype frequency**. The level of significance is set at alpha = 0.05. Each analysis is based on 1000 datasets consisting of 1000 triads with affected sons or daughters and **(A)** a single designated risk haplotype “1100” and **(B or C)** two risk haplotypes “1100” and “1101.” The risk haplotype frequency is the sum of the frequencies for the two risk-related haplotypes for scenarios **(B,C)**. **(A)** $R_{G1}^{2}=R_{G2}=R_{B}=1.5$. **(B)** for “1100” $R_{G1}^{2}=R_{G2}=R_{B}=1.5$, for “1101” $R_{G1}^{2}=R_{G2}=R_{B}=1.2$ **(C)** for “1100” $R_{G1}^{2}=R_{G2}=R_{B}=1.5$, for “1101” $R_{G1}^{2}=R_{G2}=R_{B}=1∕1.2$. The dots represent the simulated power and the curves were fit with cubic splines.

We next compared the power of the five methods under different risk scenarios, with results shown in Figure 2. In all three of these scenarios, although the risk models differed, the relationship between the 5 methods under HWE was similar. PIX-HAP performed similarly to HAPLIN. X-APL, FBAT, and UNPHASED tended to have less power and those three methods performed similarly. Scenario C was similar to scenario B except that instead of an increased risk haplotype “1101” conferred a protective effect. The powers for the latter two scenarios were fairly similar, especially for haplotype prevalence below 0.5.

### Oral cleft

**Figure 3:**
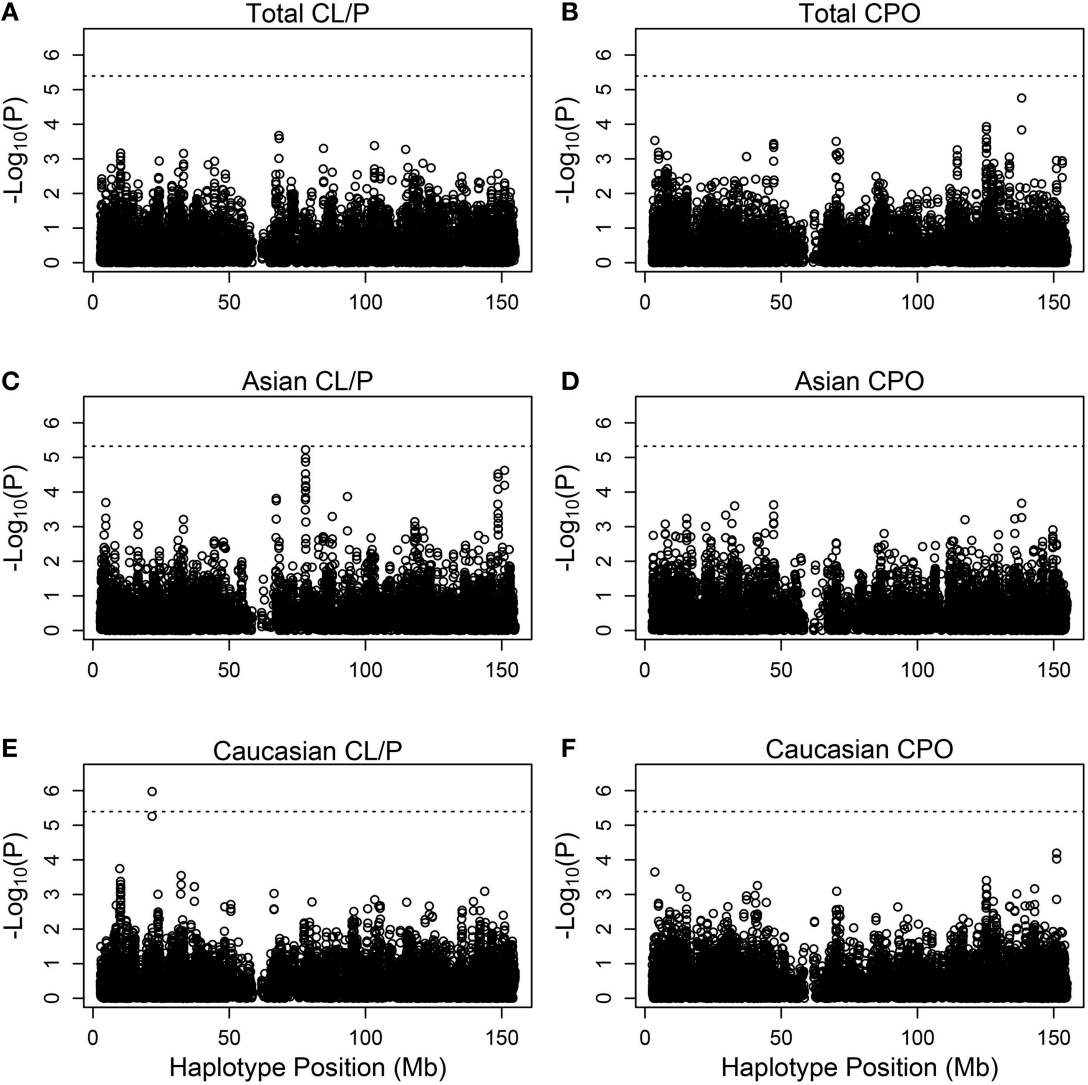
**Individual haplotype significance of the cleft examples**. The *p*-values [shown as −log10(*p*)] are calculated from PIX-HAP applied to haplotypes consisting of 4 SNPs using dbGaP data from families with oral cleft. Models were run on cleft lip with or without cleft palate families amongst **(A)** Asians and Caucasians, **(C)** Asians only, **(E)** Caucasians only, as well as cleft palate only families amongst **(B)** Asian and Caucasians, **(D)** Asians only, **(F)** Caucasians only. The dashed horizontal lines are the Bonferroni-corrected threshold for an alpha of 0.05, where the adjustment is specific to the panel of tests.

Figure 3 shows Manhattan plots for the distinct and the combined populations for the two phenotype categories. The most significant hit is for Caucasians with cleft lip with or without palate, based on (rs5904577, rs12388077, rs4317707, rs5951456), for which a 4 degree-of-freedom likelihood ratio test statistic is 33.2 (*p* = 1.06 × 10^−6^). On closer inspection of the estimated relative risks, this effect appears to be protective and driven by rare haplotypes that carry rs12388077. If we consider instead the smaller subset that includes (rs12388077, rs4317707, rs5951456), the p value is reduced to 6.1 × 10^−7^, and again the effect appears to be due to rs12388077. The effect is not seen in the Asian population, where the 4-degree-of-freedom chi-squared is close to 1.0.

We next took a closer look at the genotyping data for rs12388077 and discovered a troubling pattern, which ultimately led us to discount that finding of haplotypes containing rs12388077 as related to cleft lip with or without cleft palate. Among Caucasian fathers in complete triads who carried the variant allele at rs12388077 the genotyping for rs5951456 failed 89/93 times (Table 4). Similarly, among Caucasian sons who carried the variant allele at rs12388077, the genotyping for rs5951456 failed 45/46 times. Among mothers or daughters who carried only one copy of the variant at rs12388077, the genotyping for rs5951456 never failed (0/137 for mothers and 0/68 for daughters), but among mothers or daughters who carried two copies of that variant, the genotyping for rs5951456 always failed 9/9 times (mother) and 2/2 times (daughter). Thus homozygosity for the variant at rs12388077 for males (1 copy) or females (2 copies) was a strong predictor of genotyping failure at rs5951456 in Caucasians. The two loci are 2900 base pairs apart (by Build 37). Adding to the mystery, the same missingness dependency pattern was not seen in Asians, for whom the genotyping at rs5951456 never failed (data not shown). This observation should serve as a cautionary tale: missingness of genotype data at one locus evidently can be strongly differential by genotype at another locus. The basis for this influence is unclear; but its consequence is clear: missingness of haplotypes can be informative and produce strong bias. The relatively low call rates for rs5951456 should also have alerted us to possible bias due to genotyping errors.

**Table 4:** **A mechanism generating informative missingness for haplotypes**.

	**rs12388077**	**rs5951456**			
		**0**	**1**	**2**	**NA**
	0	741	42	−	0
Fathers	1	3	1	−	89
	0	450	32	−	1
Sons	1	0	1	−	45
	0	630	99	1	0
Mothers	1	128	3	6	0
	2	0	0	0	9
	0	251	24	2	0
Daughters	1	62	2	4	0
	2	0	0	0	2

*Number of fathers, mothers, sons and daughters who carry each combination of SNPs rs12388077and rs5951456 in complete Caucasian families. NA refers to missing genotype. 0,1,2 refer to the number of minor allele copies carried*.

There is a borderline hit in the Asian population for cleft lip with or without palate at SNPs (rs5959189, rs5959190, rs31233295, rs3132267), where the 5 degree-of-freedom chi-squared was 32 (*p* = 6 × 10^−6^). Again the effect appears to be driven by rare haplotypes that confer protection. Examination of missing genotype patterns did not reveal evidence for cross-locus influences on the genotyping–suggesting that this hit may be real. This part of the X is associated with ribosomal protein L7 pseudogene 54, and functional effects of variants are not known.

**Figure 4:**
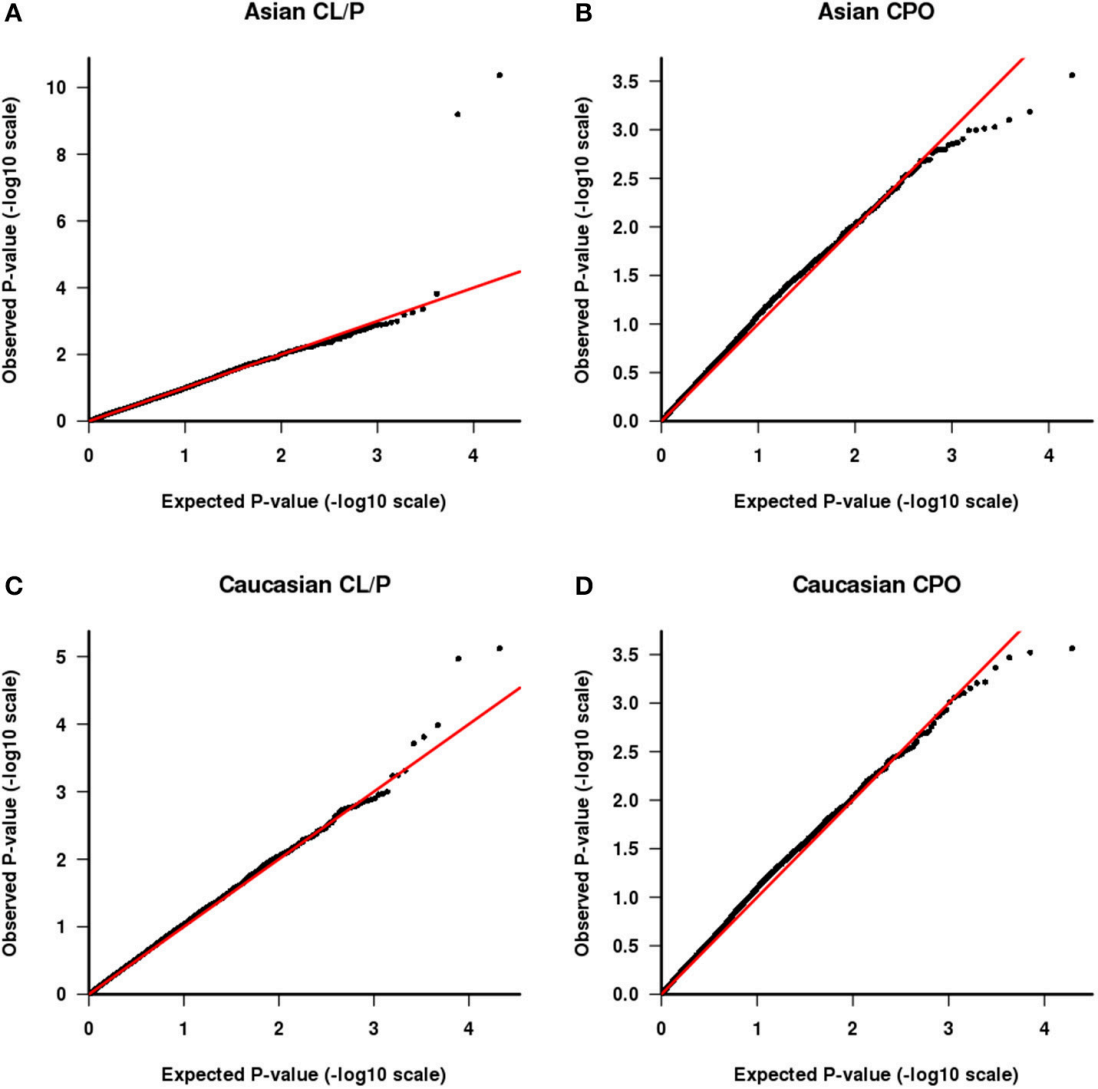
**QQ plot of −log10(***p***) as calculated from the test of parental haplotype exchangeability**. CL/P is shown for Asian families **(A)** and Caucasian families **(C)** and CPO is shown for Asian families **(B)** and Caucasian families **(D)**.

We also tested the PHE assumption, using the logistic model described above. Figure 4 shows Q-Q plots, with Asians shown in Figures 4A,B for CL/P and CPO, respectively and Caucasians shown in Figures 4C,D for the same sub-phenotypes. The two SNPs with genotyping issues identified above have been removed from the assessment for Caucasian families with CL/P. The two strong outliers seen here in Asian families affected by CL/P (Figure 4A, one haplotype involves rs6627483, rs5970136, rs5970137, and rs964180 and other overlapping haplotype involves rs12843815, rs6627483, rs5970136, and rs5970137) led us to examine those genotypes more closely, revealing another surprising anomaly: the minor allele at SNP rs5970137 was never carried by any males, but only by females. For example, among 39 families with an affected son and a heterozygous mother, no son inherited that minor allele. However, on further inspection we discovered that this pattern arose because of a genotyping problem much like that shown in Table 4. Again the relatively low call rate for rs6627483 could also have served to alert us to the potential for bias due to informative missingness. Table 5 shows the results for two SNPs demonstrating again that the genotype at one locus can affect the call rate at another locus. The genotyping issues were similar in Caucasians but SNP rs5970137 was filtered out from the analysis due to its rarity. There remain two outliers in Figure 4C.

**Table 5:** **Genotypes influencing genotypability**.

	**rs6627483**	**rs5970137**			
		**0**	**1**	**2**	**NA**
	0	698	0	−	0
Fathers	1	121	0	−	0
	NA	0	68	−	0
	0	459	0	−	0
Sons	1	70	0	−	0
	NA	1	45	−	0
	0	553	104	0	1
Mothers	1	183	0	0	0
	2	16	21	0	0
	NA	0	0	9	0
	0	181	45	0	0
Daughters	1	64	0	0	0
	2	5	12	0	0
	NA	1	0	4	0

*Number of fathers, mothers, sons, and daughters who carry each combination of SNPs rs6627483 and rs5970137 in complete Asian families with a child affected with cleft lip ± palate. NA refers to missing genotype. 0,1,2 refer to the number of minor allele copies carried. These SNPs produced the two outliers in Figure 4A*.

Two possible mechanisms unrelated to violations of PHE in the source population can cause violations to be detected by our test of PHE. First, an actual effect of the fetally-inherited haplotype(s) on risk would produce asymmetry across parents, through an etiologic mechanism. And second, there could be a maternally-mediated genetic effect that acts prenatally on the fetus to cause susceptibility to the outcome under study. Under a maternal mechanism, mothers of affected individuals will be relatively enriched for a causative haplotype, regardless of what they transmit to the affected offspring. By contrast, if the transmitted haplotype confers risk on the fetus, such a mechanism should produce asymmetry in the parents as well, but in a way that depends on the sex of the affected offspring: mothers of affected boys and fathers of affected girls will be relatively enriched for the causative haplotype. The presence of a significant effect of sex of the affected offspring in the model used for the PHE test can thus be interpreted as suggesting that it is the transmitted haplotype that affects risk. These strategies are summarized in Table 6.

**Table 6:** **Distinguishing among possible explanations for significant findings in PHE testing**.

**Other evidence in the data**	**Mechanisms consistent with the evidence**
Haplotype over-transmitted to affected offspring and…mothers of boys and fathers of girls are relatively enriched with the haplotype	Actual effect of the fetally-inherited haplotype
No evident transmission distortion and the evident asymmetry across parents in the PHE analysis does not reverse directions depending on sex of the affected offspring	Actual violation of PHE (due to nonrandom mating)^*^ or Actual maternally-mediated effect of the haplotype

* *Conclusion strengthened if exclusion of ethnically-mixed marriages removes the apparent violation of PHE*.

With this framework in mind, we next consider the two outliers in Figure 4C. The smallest p value is driven by a haplotype that is rare in fathers both of boys and of girls and also is under-transmitted to affected offspring. These observations are consistent with three possibilities: there are genotyping problems in males for this haplotype; or the haplotype is paradoxically both causative via a maternal mechanism and also protective when transmitted; or this is a chance finding. The latter seems most plausible. The second smallest *p*-value (at the following set of loci: rs4844160, rs2136826, rs1777640, rs7060899) is driven by a rare haplotype that was never found in a father but was transmitted from the mother in a Mendelian way, both to boys and girls. We consider this likely to be a chance finding.

## Discussion

For family-based studies, our simulations suggest that the method we have proposed, PIX-HAP, offers slightly better power than the other available approaches for studying associations between diseases and haplotypes on the X chromosome. It also offers robustness against bias due to violations of HWE, as does HAPLIN when the data are complete. The exchangeability assumption we require, PHE, is weaker than HWE, thereby allowing broader valid application, while improving power by taking advantage of information embedded in the distribution of haplotypes across parents. It also enables estimation of the risk ratios associated with haplotypes and the estimation can be done separately for male and female offspring.

Our method relies on an exchangeability assumption, which can be assessed using the data. Apparent violations of PHE require additional exploratory analysis, as outlined in Table 6. The kind of comparative approach specified in row 3 of Table 6 cannot be used to disentangle maternal from offspring effects if the defect only occurs in one sex, such as crypt-orchidism. For such an outcome, one would have to test separately for asymmetry in the parents versus over-transmission of the causative haplotype from heterozygous mothers to affected boys.

PHE can also fail for reasons unrelated to etiology. In addition to the genotyping dependencies noted in Tables 4, 5, if the population is subject to asymmetric mating, e.g., because it is more common for Caucasian women to marry African-American men than the other way around, then PHE (and HWE) could be violated. In such a circumstance, one could restrict the analysis to only use families with ethnically matched parents, e.g., based on principal components. One could augment the families with ethnically similar parents by also including maternal transmissions for the ethnically unmatched couples who have an affected son (only using one pseudo-brother for those sons), thereby bypassing the paternal information.

The genotyping anomaly we discovered highlights the need to look very closely at haplotypes that appear to show evidence of association. The ability to genotype a given SNP can evidently depend on the observed genotype at a nearby SNP, causing haplotypes to be informatively missing. Presumably the technology has improved since the clefting genotyping was done for this project.

While our simulations were all based on simplex families with one affected offspring and two parents, FBAT can handle multiplex families. PIX-HAP could also do that, by inclusion of multiple cases in the conditional logistic analysis.

The method as we described it does not allow inclusion of incomplete triads, e.g., where a father may be missing. However, inclusion of families with a mother and affected son would be straightforward, by only allowing the one pseudo-son corresponding to the nontransmitted maternal X, as mentioned above. Similar inclusion of mother-daughter families would be much harder because of phase ambiguity. Potentially, inclusion of data from such families would be possible by application of the expectation-maximization (EM) algorithm, but one would either need to assume HWE or to have a study large enough to estimate a large number of parental mating type parameters. Such a study could profitably genotype siblings (ideally a brother) of the affected daughters to better identify the possible paternal X haplotypes. However, such extensions are beyond the scope of the current paper.

## Author contributions

This research was carried out as part of Dr. AW's dissertation work for the Biostatistics Department at the University of North Carolina at Chapel Hill, for which she was awarded the Doctor of Public Health degree last spring. She was a predoctoral IRTA Fellow at NIEHS during the research. Her mentors were Dr. CW and Dr. MS. Dr. CW contributed ideas and direction. Drs. MS and AW contributed equally to the paper.

### Conflict of interest statement

The authors declare that the research was conducted in the absence of any commercial or financial relationships that could be construed as a potential conflict of interest.
